# Current Status of Sodium Bicarbonate in Coronary Angiography: An Updated Comprehensive Meta-Analysis and Systematic Review

**DOI:** 10.1155/2015/690308

**Published:** 2015-04-21

**Authors:** Sadegh Ali-Hassan-Sayegh, Seyed Jalil Mirhosseini, Elham Rahimizadeh, Zahra Ghodratipour, Zahra Sarrafan-Chaharsoughi, Ali Mohammad Dehghan, Mohammad Reza Lotfaliani, Mohammad Rezaeisadrabadi, Elham Kayvanpour, Farbod Sedaghat-Hamedani, Mohamed Zeriouh, Alexander Weymann, Anton Sabashnikov, Aron-Frederik Popov

**Affiliations:** ^1^Cardiovascular Research Center, Shahid Sadoughi University of Medical Sciences, Yazd, Iran; ^2^Department of Medicine III, University of Heidelberg, Heidelberg, Germany; ^3^Department of Cardiothoracic Transplantation and Mechanical Circulatory Support, Royal Brompton & Harefield NHS Foundation Trust, London, UK

## Abstract

This systematic review with meta-analysis sought to determine comparison of efficacy and safety of hydration with sodium bicarbonate versus sodium chloride on contrast induced nephropathy and clinical outcomes. We searched major electronic databases for studies in randomized controlled trials. A value of *P* < 0.1 for Q test or *I* ^2^ > 50% indicated significant heterogeneity between the studies. Literature search of all databases retrieved 650 studies. 29 studies enrolled in meta-analysis. Pooled analysis indicated about the incidence of CIN (OR of 0.718; 95% CI: 0.60 to 0.85; *P* = 0.000), requirement of hemodialysis (OR of 1.00; 95% CI: 0.49 to 2.01; *P* = 0.9), mean changes of serum creatinine (WMD of 2.321; 95% CI: 1.995 to 2.648; *P* = 0.000), length of hospital stays (WMD of −0.774; 95% CI: −1.65 to 0.10; *P* = 0.08), major adverse cardiovascular events (OR = 1.075, 95% CI: 0.59 to 1.95; *P* = 0.8), and mortality (OR of 0.73; 95% CI: 0.42 to 1.26; *P* = 0.2). Overall, hydration with sodium bicarbonate could significantly reduce CIN and the length of hospital stay compared to sodium chloride. In addition NAC added as a supplement to sodium bicarbonate could increase prophylactic effects against nephropathy.

## 1. Introduction

The contrast-induced nephropathy (CIN) is the third most common cause of acute renal failure (ARF) worsening in preexisting renal function impairment that has been associated with morbidity, mortality, and prolonged hospitalization as well as increase in therapy costs [1, 2]. Diagnosis of CIN is based upon acute impairment of renal function reflected by an absolute increase in the serum creatinine concentration of 0.5 mg/dL or by relative elevation as >25% of baseline within 2 days of contrast exposure [3, 4]. Chronic renal failure, diabetic mellitus, contrast media volume, and recurrent administration are considered impairment risk factors [3, 4]. The pathogenesis of CIN involves a combination of insults affecting renal tubular endothelial cells such as intrarenal vasoconstriction and ischemia, reperfusion injury, and toxicity of renal cells [5]. Currently, the standard of care in the management of patients who require coronary diagnostic imaging is enough hydration, minimizing the volume of contrast agent, and careful use of nephrotoxic drugs [6, 7]. Hydration could not only increase renal blood flow and reduce renal vasoconstriction, generation of renal vasoconstrictor substance and renal medullary ischemia but also directly reduce the toxicity of contrast agent and incidence of CIN [8]. Recently, studies have begun to evaluate whether volume supplementation with sodium bicarbonate may be superior to volume supplementation with sodium chloride 0.9% [9, 10]. Sodium bicarbonate can decrease the formation of reactive free oxygen radicals by increasing pH and producing renal protective markers [9, 10]. The ideal hydration regimen for preventing CIN remains undefined.

This systematic review with meta-analysis sought to determine the strength of evidence for comparison of effects of sodium bicarbonate versus sodium chloride on incidence of CIN, requirement of hemodialysis, level of serum creatinine, and mortality after coronary angiography.

## 2. Methods and Materials

### 2.1. Literature Search

A comprehensive literature search was conducted in major electronic databases (Medline/Pubmed, Embase, Elsevier, Web of Knowledge, Sciences online database, and Google Scholar) from their inception through August 16, 2014, to identify RCTs that reported comparison of effects of sodium bicarbonate versus sodium chloride on incidence of CIN, requirement of hemodialysis, heart failure, mortality, duration of hospital stay, and levels of serum creatinine, sodium bicarbonate, and potassium. Predefined search terms included “sodium bicarbonate” and “sodium chloride” and “contrast-induced nephropathy,” “CIN,” “serum creatinine,” “coronary angiography,” and “coronary imaging.” No language restrictions were applied. All retrieved references of the included RCTs were also reviewed to determine additional studies not indexed in common databases. Studies were included into the analysis when they met the following criteria: (1) RCT, (2) comparison of hydration of sodium bicarbonate with a control group, and [4] reporting data on the incidence of radiocontrast-induced complications according to our review-checklist. Congress presentation and abstracts without peer-reviewed publications of manuscripts were not included in this review.

### 2.2. Data Extraction and Outcome Measures

Two investigators (Sadegh Ali-Hassan-Sayegh, Elham Rahimizadeh) extracted the data independently, and discrepancies were resolved via a consensus standardized abstraction checklist used for recording data in each study. Data retrieved from the trials included author's name, type of radiocontrast (low-, iso-, or high-osmolality), details of hydration regimens, mean baseline serum creatinine, study design, sample size, mean age, and gender. The incidence of CIN, requirement of hemodialysis, incidence of heart failure and mortality, duration of hospital stay, and levels of serum creatinine, sodium bicarbonate, and potassium were recorded for each group. For exploration of heterogeneity among trials, subgroup analysis of disparities in the patients' characteristics was performed for (1) average age (<65 versus ≥65 years), (2) diabetes (≤30 versus >30%), (3) radiocontrast (low, iso), and (4) procedure (elective versus emergency).

### 2.3. Definitions

CIN is defined as ≥25%, ≥0.5 mg/dL, ≥25%, and ≥0.5 mg/dL increase in creatinine from baseline and renal failure is defined as new onset of hemodialysis.

### 2.4. Statistical Analysis, Publication Bias, and Quality Assessment

Data were analyzed by STATA version 11.0 utilizing METAN and METABIAS modules. The effect sizes measured were odds ratio (OR) with 95% confidence interval (CI) for categorical variable. For noncategorical data the weighted mean difference (WMD) with 95% CI was used for calculating differences in serum creatinine, sodium bicarbonate, and potassium and length of hospital stay between sodium bicarbonate and sodium chloride groups. OR < 1 favored sodium bicarbonate and OR > 1 favored sodium chloride. RCTs with no events in the 2 arms were discarded from pooled analysis. Forest plots were created for each outcome. A value of *P* < 0.1 for *Q* test or *I* ^2^ > 50% indicated significant heterogeneity among the studies. Heterogeneity among trials was accounted for by applying a random effect model when indicated. The presence of publication bias was evaluated using the Begg and Egger tests. Quality assessment of RCTs was performed by using the Jadad score. The Jadad score assesses 3 items including randomization (0–2 points), blinding of study (0–2 points), and withdrawals and dropouts (0-1 points). Higher scores indicate better reporting (“high” quality: 5; “good” quality: 3-4; “poor” quality: 0–2). Results were considered statistically significant at a *P* value less than 0.05.

## 3. Results

### 3.1. Literature Search Strategy and Included Trials

**Figure 1:**
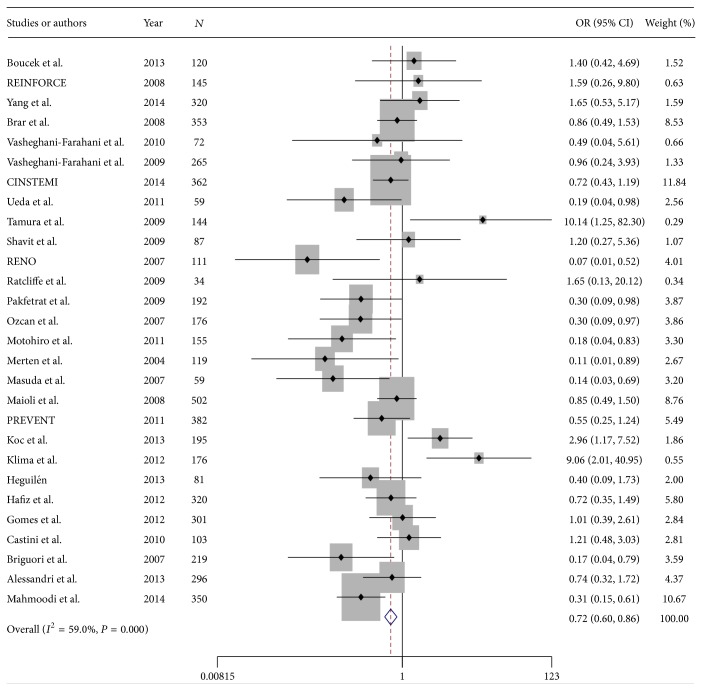
Forest plot of odds ratio (OR) for hydration with sodium bicarbonate on contrast-induced nephropathy.

Literature search retrieved 650 studies from screened databases of which 400 (61.5%) were excluded after initial review (Figure 1). Of 250 primary included studies, 221 were excluded after detailed evaluation due to insufficient reporting of endpoints of interest. The final analysis included 29 RCTs.

### 3.2. Study Characteristics and Effect Measures (Sodium Bicarbonate versus Sodium Chloride)

#### 3.2.1. Incidence of Contrast-Induced Nephropathy

A total of 5698 patients were included from 28 RCTs reported data about effects of hydration with sodium bicarbonate on incidence of CIN (Table 1). Patient population of RCTs ranged from 34 to 502 patients. From 5698 patients, 2847 cases were allocated to SB and 2851 cases to the SC group. The overall incidence of CIN was 10.03% ranging from 3.01% to 23.3%. CIN occurred in 8.57% in SB group and 11.50% in SC group (Table 2). Pooled treatment effect analysis revealed that SB versus SC significantly reduced the incidence of CIN with an OR of 0.718 (95% CI: 0.60–0.85; *P* = 0.000) using a random model (Figure 1). Significant heterogeneity was observed among the RCTs (chi-squared = 65.79, *I* ^2^ = 59.0%). Subgroup analysis showed that preventing effects of SB was superior to SC on CIN in patients aged above and below 65 years, diabetic and nondiabetic patients, cases undergoing low-osmolality radiocontrast imaging compared to iso-osmolality and cases undergoing emergency angiography compared to elective angiography (Table 3). Begg and Egger tests showed that there was no potential publication bias among the included RCTs (Begg test, *P* = 0.327; Egger test, *P* = 0.327).

#### 3.2.2. Requirement of Hemodialysis

**Figure 2:**
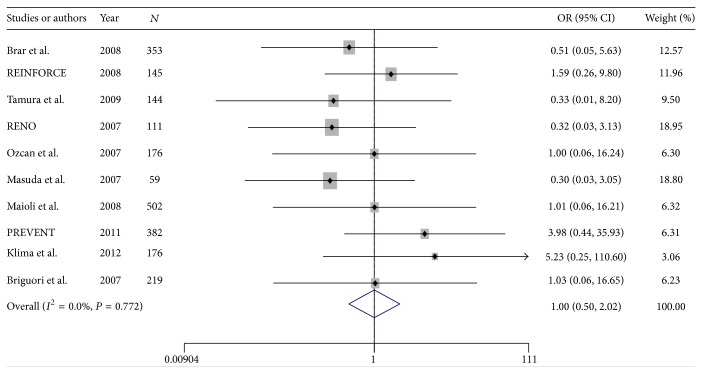
Forest plot of odds ratio (OR) for hydration with sodium bicarbonate on incidence of requirement dialysis.

A total of 3765 patients were included from 19 RCTs reporting data on requirement of hemodialysis (Table 1). After removing RCTs with no events in 2 arms, a total of 2267 patients were included from 10 studies enrolled in meta-analysis. From all patients, 1130 cases were allocated to SB and 1137 to the SC group. The overall incidence of requirement of hemodialysis was 1.32% ranging from 0.3% to 6.7%. Requirement of hemodialysis occurred in 1.32% of the cases in SB group and 1.31% in control group (Table 2). Pooled treatment effect analysis revealed that SB versus SC could not reduce incidence of requirement of hemodialysis after coronary angiography with an OR of 1.00 (95% CI: 0.49–2.01; *P* = 0.9) using a fixed model (Figure 2). No significant heterogeneity was observed among the RCTs (chi-squared = 5.67, *I* ^2^ = 0.0%).

#### 3.2.3. Mean Changes of Serum Creatinine, Sodium Bicarbonate, and Potassium

**Figure 3:**
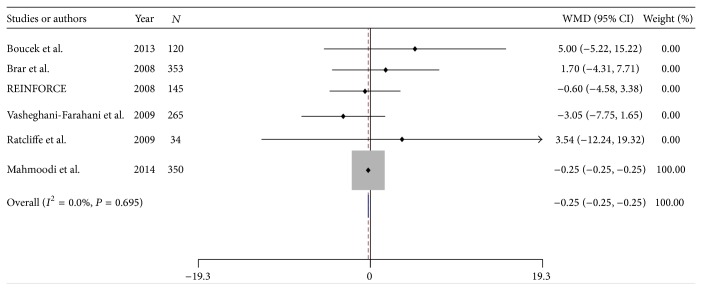
Forest plot of weighted mean differences (WMD) for hydration with sodium bicarbonate on mean changes of creatinine.

From 1267 patients, 636 cases were allocated to SB and 631 to the SC group (Table 1). Pooled analysis showed that SB had similar effects on mean serum creatinine with WMD of −0.250 (95% CI: −0.254 to −0.246; *P* = 0.000) using a fixed model (Figure 3). No significant heterogeneity was observed among the RCTs (chi-squared = 3.03, *I* ^2^ = 0.0%). Mean level of serum sodium bicarbonate for 5 trials was 0.46 ± 3.28 with 1.77 ± 2.93 for SB and −0.85 ± 3.63 for the SC group. From 891 patients, 448 cases were allocated to SB and 443 to the SC group (Table 1). Pooled analysis showed that SB versus SC could significantly increase mean level of serum sodium bicarbonate with WMD of 2.321 (95% CI: 1.995 to 2.648; *P* = 0.000) using a random model (see Supplemental Figure  1 in Supplementary Material available online at http://dx.doi.org/10.1155/2015/690308). Significant heterogeneity was observed among the RCTs (chi-squared = 23.58, *I* ^2^ = 83%). A total of 656 patients were included from 3 RCTs reporting data on mean level of serum potassium (Table 1). Pooled analysis indicated that SB versus SC could significantly decrease mean level of serum potassium with WMD of −0.091 (95% CI: −0.171 to −0.011; *P* = 0.02) using a fixed model (Supplemental Figure  2).

#### 3.2.4. Length of Hospital Stays (LHS)

Mean LHS for 5 trials (817 patients) were 8.47 ± 9.71 days with 8.24 ± 9.76 for SB and 8.7 ± 9.66 for the SC group (Tables 1 and 2). Pooled analysis revealed that SB had a trend towards decreasing LHS with a WMD of −0.774 (95% CI: −1.65 to 0.10; *P* = 0.08) using a random effect model. No Significant heterogeneity was observed among the RCTs (chi-squared = 7.58, *I* ^2^ = 47.2%).

#### 3.2.5. Major Adverse Cardiovascular Events (MACE)

After discarding 5 RCTs for having no events in 2 comparative arms, 4 RCTs (914 patients) were included in the analysis. Major adverse cardiovascular events occurred in 5.66% of the cases in SB group and 5.27% in SC group (Tables 1 and 2). Pooled treatment effect analysis demonstrated that incidence of MACE is similar in SB and SC groups (OR = 1.075, 95% CI: 0.59–1.95; *P* = 0.8, and chi-squared = 4.77, *I* ^2^ = 37.1%) (Supplemental Figure  3).

#### 3.2.6. Mortality

After discarding 7 RCTs because of no death event in 2 comparative arms, 8 RCTs were used for the meta-analysis. Mortality occurred in 2.26% in SB and 3.08% in SC group (Tables 1 and 2). Pooled treatment effect analysis showed that SB versus SC could not significantly reduce incidence of mortality with an OR of 0.73 (95% CI: 0.42–1.26; *P* = 0.2) using a fixed model. No significant heterogeneity was observed among the RCTs (chi-squared = 7.43, *I* ^2^ = 5.8%) (Supplemental Figure  4).

### 3.3. Sodium Bicarbonate versus Sodium Bicarbonate Plus Anti-Oxidant Agent (N-Acetyl Cysteine)

#### 3.3.1. Incidence of Contrast-Induced Nephropathy

**Figure 4:**
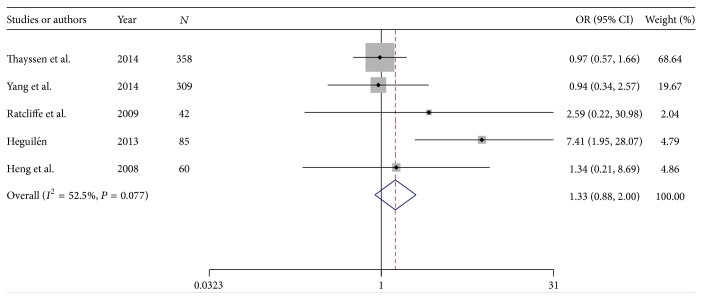
Forest plot of odds ratio (OR) for treatment with N-acetyl cysteine and hydration with sodium bicarbonate on contrast-induced nephropathy.

A total of 5 RCTs (854 patients) were used for the analysis. Patient population of the RCTs ranged from 42 to 358 patients. From 854 patients, 433 cases were allocated to SB alone and 421 to the SB plus NAC group. The overall incidence of CIN was 12.67% ranging from 5.17% to 21.17%. CIN occurred in 14.08% in SB alone and 11.16% in SB plus NAC group. Pooled treatment effect analysis revealed that SB plus NAC versus SB alone had trend towards reducing the incidence of CIN with an OR of 1.32 (95% CI: 0.87–1.99; *P* = 0.1) using a random model. Significant heterogeneity was observed among the RCTs (chi-squared = 8.42, *I* ^2^ = 52.5%) (Figure 4).

## 4. Discussion

CIN occurs in more than 15% of patients with chronic renal impairment undergoing diagnostic and therapeutic radiographic procedure [1, 2]. Approximately 0.5% to 12% of these patients require dialysis and longer length of hospital stay, accompanied by worsening of renal function, possibly expediting the evolution toward end stage renal failure [1–4]. Several protocols have been introduced for prevention of CIN including: periprocedural hydration with isotonic or hypotonic saline and antioxidant compounds such as N-acetyl cysteine (NAC) or ascorbic acid, hemofiltration [2–5]. Regarding the fact that production of free radical oxygen is known as one of the most important pathogenesis of CIN, sodium bicarbonate with its alkali nature might be effective in prevention of CIN [9, 10]. The present study revealed that volume expansion with sodium bicarbonate infusion could stimulate dilution of circulating contrast medium and vasoconstrictive mediators and prevent activator of tubuloglomerular feedback and had preventing effect on the incidence of CIN more than normal saline hydration. The results of subgroup analysis indicated that hydration with sodium bicarbonate could reduce the incidence of CIN in both diabetic and nondiabetic patients. Also hydration with sodium bicarbonate showed to be more effective in emergency coronary imaging and high-risk patients as compared with elective coronary imaging. Jang et al. found sodium bicarbonate in remarkable preference to sodium chloride in prevention of CIN. They also reported that patients undergoing emergency imaging would receive more prophylactic effects compared to elective procedures [11]. Possibly acid-base and electrolyte imbalances in high-risk patients undergoing emergency coronary imaging would intensify following toxic effects of radiocontrast and sodium bicarbonate as an antiacid is likely to better control acidic conditions. Antioxidants reduce free radical oxygen, thus being recommended as appropriate therapeutic supplements [12]. The findings of our study suggested that NAC added as an antioxidant supplement to sodium bicarbonate had trend towards reducing the incidence of CIN more than sodium bicarbonate alone. Brown et al. reported that NAC plus sodium bicarbonate hydration could have more protective effect on renal function compared to hydration alone [12]. Subgroup analysis revealed that the least incidence of CIN was when the patients underwent low-osmolality radiocontrast angiography and adequate hydration with sodium bicarbonate plus NAC. Our findings confirmed that iso-osmolality radiocontrast, in comparison with low-osmolality, increased the incidence of CIN; therefore, increase in osmolality intensified acute renal failure. We could assume that the beneficial effects of sodium bicarbonate might be offset by low-osmolar contrast medium volume, which had a more physiologic profile in terms of renal hemodynamics. CIN following angiography could predispose the incidence of renal failure and requirement of dialysis in high-risk patients with diabetic nephropathy and heart failure [3–8]. Despite low incidence of requirement of dialysis, it is of high importance because the patients with this complication generally become prone to morbidity, decrease in quality of life, need for renal transplantation, and mortality. Hydration with sodium bicarbonate had no preference for decreasing hemodialysis and mortality in comparison with sodium chloride. Several previous investigations indicated that clinical endpoint such as renal replacement therapy, heart failure, and mortality were not improved following hydration with sodium bicarbonate versus sodium chloride [11, 12]. This result may be explained by the fact that the patients who require dialysis in the period of follow-up, in addition to a history of renal disease before angiography, renal cells toxicity is too severe after exposure to radiocontrast leading to crisis of symptoms and incidence of severe CIN [8, 9]. This condition is malignant to such an extent that change in hydration or even addition of drug supplements cannot have protective effects. Acute nephropathy after angiography could increase the length of hospital stay. The current study revealed that hydration with sodium bicarbonate had more trend of decreasing hospitalization compared to sodium chloride. This decrease might be due to reduction in the incidence of CIN and cares related to renal disorders.

A number of studies have found that changes in creatinine levels within 24 to 48 hours after exposure to radiocontrast could be considered as equivalent indicator for new onset CIN [13–18]. Therefore, an increase of 0.5 mg/dL after angiography demonstrates the incidence of an acute nephropathy. The effects of sodium bicarbonate and sodium chloride on the mean changes of creatinine were not significantly different. Our findings found elevated serum sodium bicarbonate and decreased serum potassium after hydration with sodium bicarbonate. Therefore, in hydration with sodium bicarbonate, patients condition should be monitored regarding acid-base balance and changes in electrolytes levels. Finally it is concluded that hydration with sodium bicarbonate could significantly reduce CIN and the length of hospital stay compared to sodium chloride. In addition NAC added as a supplement to sodium bicarbonate could increase prophylactic effects against nephropathy. It is recommended to regularly monitor the patients following hydration with sodium bicarbonate regarding acid-base balance and changes in potassium level in order to avoid complications.

## Supplementary Material

Supplementary Figure 1
Supplementary Figure 2
Supplementary Figure 3
Supplementary Figure 4

## Figures and Tables

**Table 1:** Demographic data of included studies.

Author	*N*		Mean age (years)		Male (%)		Regimen	Contrast media	Mean baseline sCr
	SB	SC	SB	SC	SB	SC			
Sodium bicarbonate versus saline									

Boucek et al. [13]	61	59	63	67	75.4	74.5	SB solution was produced by adding 154 mL of 8.4% NaHCO_3_ to 846 mL of 5% glucose. 1 h immediately before (at the rate of 3 mL/kg BW/h limited the maximal amount 330 mL and for 6 h following the intervention	Nonionic low-osmolar contrast medium	119 micromol per litter

Brar et al. [14]	175	178	71	71	62	65	Infusion was begun 1 h prior to the start of contrast administration at 3 mL/kg for 1 h and decreased to 1.5 mL/kg per h during the procedure and for 4 h following completion of the procedure	Nonionic low-osmolar contrast medium	1.5 mg/dL

Alessandri et al. [19]	138	158	64	65	66.6	67.7	160 mEq of NaHCO_3_ in 350 mL of 5% glucose solution 2 mL/kg/h since two hours before the administration of contrast medium. The infusion prolonged for the following six hours after the procedure with an infusion rate of 1 mL/kg/h	Nonionic low-osmolality contrast medium	1.5 mg/dL

REINFORCE trial [15]	71	74	70.1	72.7	74.6	81	154 mEq of 1000 mEq/L of SB in 5% dextrose solution, prepared at the hospital pharmacy by adding 154 mL of 1000 mEq of SB to 846 mL of 5% dextrose solution and adjusting the dextrose concentration to 4.23%. Fluids were administrated IV at the rate of 2 mL/kg of body weight per hour for 2 h before, at the rate of 1 mL/kg of body weight per hour during, and 6 h after administration of contrast medium	Nonionic iso-osmolar contrast medium	1.6

Yang et al. [20]	159	161	58.7	59.6	52.8	53.4	SB intravenously infused with 1.5% SB solution at the rate of 1.5 mL/kg/h 6 h before the application of the contrast agent. After the contrast exposure was applied the 1.5% SB infusion was continued for 6 h.	Nonionic low-osmolality contrast medium	70 micromol per litter

Vasheghani-Farahani et al. [21]	36	36	61.4	62.7	77.7	80.5	75 mL of 8.4% SB to 1 liter of 0.45% SC. Intravenous bolus was given at the rate of 3 mL/kg for 1 h immediately before contrast injection, followed by an infusion of 1 mg/kg per hours for 6 h after the procedure	Low-osmolar contrast medium iohexol	1.7

Vasheghani-Farahani et al. [16]	135	130	62.9	63.8	91.4	81.5	75 mL of 8.4% SB to 1 liter of 0.45% SC. Intravenous bolus was given at the rate of 3 mL/kg for 1 h immediately before contrast injection, followed by an infusion of 1 mg/kg per hours for 6 h after the procedure	Low-osmolar contrast medium iohexol	1.6

CINSTEMI trial [22]	181	181	62	63	76.8	80.1	167 (mmol/L) SB intravenously as 500 mL in the first hour followed by infusion of 100 mL per hour in the next 5 hours	Nonionic iso-osmolar contrast medium	0.8

Ueda et al. [23]	30	29	77	75	77	79	0.5 mg/kg SB as soon as possible after hospital admission and 1 mL/kg/h during and 6 hours after the procedure	Low-osmolar contrast medium	1.4

Tamura et al. [24]	72	72	72.3	73.3	91.7	83.3	20 mEq SB 5 min before contrast exposure	Low-osmolar contrast medium iohexol	1.3

Shavit et al. [25]	51	36	72	71	84	70	154 mEq per liter SB in 5% dextrose in water mixed by adding 154 mL of the 1000 mEq per liter SB to 846 mL of 5% dextrose in water. The initial IV bolus was 3 mL/kg for 1 h before procedure and 1 mL/kg per liter for 6 hours after procedure	Low-osmolar contrast medium	1.8

RENO trial [26]	56	55	65	64	68	71	Initial IV bolus 5 mL/kg/h SB with 154 mEq per liter NaHCO_3_ in 5% glucose and H_2_O, after contrast same solution continued at 1.5 mL/kg/h for 12 h the day after contrast	Nonionic low-osmolality contrast medium	1.0

Ratcliffe et al. [17]	19	15	67	64	58	60	154 mEq per liter SB in 5% dextrose in water mixed by adding 154 mL of the 1000 mEq per liter SB to 846 mL of 5% dextrose in water. The initial IV bolus was 3 mL/kg for 1 h before procedure and 1 mL/kg per liter for 6 hours after procedure	Nonionic iso-osmolar contrast medium	106 micromol per liter

Pakfetrat et al. [27]	96	96	57.8	58.5	58.3	64.5	SB solution was prepared in the hospital pharmacy by adding 154 mL 1000 mEq per liter SB to 846 mL 5% dextrose in water and was infused at 3 mL/kg/h starting 1 h before contrast administration, followed by a 1 mL/kg/h infusion for 6 h after procedure	Nonionic iso-osmolar contrast medium	1.1

Ozcan et al. [28]	88	88	68	70	72.7	75	SB solution was prepared in the hospital pharmacy by adding 154 mL 1000 mEq per liter SB to 846 mL 5% dextrose in water and was infused at 1 mL/kg/h starting 6 h before contrast administration, followed by a 1 mL/kg/h infusion for 6 h after procedure	Ionic low-osmolality contrast medium	1.3

Motohiro et al. [29]	78	77	71	74	76	64	SB solution was prepared in the hospital pharmacy by adding 154 mL 1000 mEq per liter SB to 846 mL 5% dextrose in water and was infused at 1 mL/kg/h continued from 3 h before to 6 h after procedure	Nonionic low-osmolality contrast medium	1.5

Merten et al. [30]	60	59	66.7	69.2	73	76	SB solution was prepared in the hospital pharmacy by adding 154 mL 1000 mEq per liter SB to 846 mL 5% dextrose in water. The initial IV bolus was 3 mL/kg/h for 1 h immediately before radiocontrast injection. Following this, same fluid at a rate of 1 mL/kg/h during the contrast exposure and 6 h after the procedure	Nonionic low-osmolality contrast medium	1.7

Masuda et al. [31]	30	29	75	76	63	59	154 mL SB. Intravenous bolus was given at the rate of 3 mL/kg for 1 h before contrast injection, followed by an infusion of 1 mg/kg per hours during and 6 h after the procedure	Nonionic low-osmolality contrast medium	1.3

Maioli et al. [32]	250	252	74	74	57.2	60.7	154 mL SB. Intravenous bolus was given at the rate of 3 mL/kg for 1 h before contrast injection, followed by an infusion of 1 mg/kg per hours 6 h after the procedure	Nonionic iso-osmolar contrast medium	1.2

PREVENT trial [33]	193	189	68.5	67.5	70.5	71.4	154 mL SB. Intravenous bolus was given at the rate of 3 mL/kg for 1 h before contrast injection, followed by an infusion of 1 mg/kg per hours during and 6 h after the procedure	Nonionic iso-osmolar contrast medium	1.5

Koc et al. [34]	94	101	62	62	58	48	154 mL 1000 mEq per liter SB to 846 mL 5% dextrose in water and was infused at 1 mL/kg/h starting 6 h before contrast administration, followed by a 1 mL/kg/h infusion for 6 h after procedure	Nonionic iso-osmolar contrast medium	1.0

Klima et al. [35]	87	89	78	75	66	62	The initial IV bolus SB was 3 mL/kg/h of 166 mEq per liter for 1 h immediately before injection. Following this, same fluid at a rate of 1 mL/kg/h during the contrast exposure and for 6 h after the procedure. SB 166 mEq as a bolus administrated over 20 min immediately before contrast. Additionally, oral SB (500 mg NaHCO_3_ per capsule: 1 capsule/10 kg) at the start of infusion and after contrast within 6 h	Nonionic iso-osmolar contrast medium	137 micomol per liter

Heguilén et al. [36]	43	38	67.7	69.3	62.7	78.9	154 mEq per liter of SB in 5% dextrose in H_2_O, mixed by adding 77 mL of 1000 mEq per liter SB to 423 mL of 5% dextrose in H2O and was infused at 3 mL/kg/h from at least 2 h pervious to procedure and 1 mL/kg/h during and for the next 6–12 h	Nonionic low-osmolality contrast medium	1.5

Hafiz et al. [37]	159	161	74	73	56.6	57.1	159 mEq per liter SB to 5% dextrose in water and was infused at 3 mL/kg/h starting 1 h before contrast administration, followed by a 1 mL/kg/h infusion for 6 h after procedure	Nonionic low-osmolality contrast medium	1.6

Gomes et al. [38]	150	151	64.1	64.5	69.3	74.8	154 mEq per liter SB to 5% dextrose in water and was infused at 3 mL/kg/h starting 1 h before contrast administration, followed by a 1 mL/kg/h infusion for 6 h after procedure	Ionic low-osmolality contrast medium	1.5

Castini et al. [39]	52	51	70	72.7	85	84	IV bolus administration of SB at a rate of 1 mL/kg body weight per hour for 12 hours before and 12 hours after contrast injection	Nonionic iso-osmolar contrast medium	1.5

Briguori et al. [40]	108	111	70	71	88	81	SB solution was prepared in the hospital pharmacy by adding 154 mL 1000 mEq per liter SB to 846 mL 5% dextrose in water. The initial IV bolus was 3 mL/kg/h for 1 h immediately before radiocontrast injection. Following this, same fluid at a rate of 1 mL/kg/h during the contrast exposure and 6 h after the procedure	Nonionic iso-osmolar contrast medium	2

Mahmoodi et al. [18]	175	175	64.9	64.4	43.4	59.4	Sodium bicarbonate solution was prepared by adding 154 mL of 1000 mEq/L sodium bicarbonate to 846 mL of 5% dextrose with water. All the patients received a fixed dose of fluid 6 h before the procedure and 6 h after it	Nonionic low-osmolality contrast medium	1.1

Author	*N*		Mean age (years)		Male (%)		Regimen on NAC	Contrast media	Mean baseline sCr
	SB	SB + NAC	SB	SB + NAC	SB	SB + NAC			

Sodium bicarbonate versus sodium bicarbonate plus N-acetyl cysteine									

Thayssen et al. (CINSTEMI trial) [22]	181	177	63	63	80.1	78.5	1200 mg NAC orally before procedure, followed by 1200 mg daily during the next 48 hours	Nonionic iso-osmolar contrast medium	0.8

Yang et al. [20]	159	150	59.6	60	52.8	54.67	600 mg NAC orally twice daily, at 24 h before and after procedure	Nonionic low-osmolality contrast medium	72 micromol per liter

Ratcliffe et al. [17]	19	23	64	65	60	70	1200 mg NAC intravenous bolus at 1 h before and 1200 mg orally twice daily for 48 h after procedure	Nonionic iso-osmolar contrast medium	103.3 micromol per liter

Heguilén et al. [36]	42	43	67.7	64.8	64.2	74.4	600 mg NAC orally twice daily, at 24 h before and after procedure	Nonionic low-osmolality contrast medium	1.5

Heng et al. [41]	32	28	N.D	N.D	N.D	N.D	1200 mg NAC twice daily at 24 h before and during procedure	N.D	N.D

**(a):** 

Author	CIN		Hemodialysis		Changes of serum levels of creatinine		Changes of serum levels of sodium bicarbonate	
	SB	SC	SB	SC	SB	SC	SB	SC
Sodium bicarbonate versus saline								
Boucek et al. [13]	7	5	0	0	14 ± 31	9 ± 26	N.D	N.D
Brar et al. [14]	26	30	1	2	11 ± 30.6	9.3 ± 26.9	N.D	N.D
Alessandri et al. [19]	10	15	N.D	N.D	N.D	N.D	N.D	N.D
REINFORCE trial [15]	3	2	3	2	1.7 ± 12.6	2.3 ± 11.8	N.D	N.D
Yang et al. [20]	8	5	N.D	N.D	N.D	N.D	N.D	N.D
Vasheghani-Farahani et al. [21]	1	2	N.D	N.D	N.D	N.D	N.D	N.D
Vasheghani-Farahani et al. [16]	4	4	N.D	N.D	−3.35 ± 21	−0.3 ± 18	N.D	N.D
CINSTEMI trial [22]	33	43	0	0	N.D	N.D	N.D	N.D
Ueda et al. [23]	2	8	0	0	N.D	N.D	N.D	N.D
Tamura et al. [24]	9	1	0	1	N.D	N.D	N.D	N.D
Shavit et al. [25]	5	3	0	0	N.D	N.D	N.D	N.D
RENO trial [26]	1	12	1	3	N.D	N.D	N.D	N.D
Ratcliffe et al. [17]	2	1	N.D	N.D	14.14 ± 12.38	10.6 ± 29.1	N.D	N.D
Pakfetrat et al. [27]	4	12	0	0	N.D	N.D	N.D	N.D
Ozcan et al. [28]	4	12	1	1	N.D	N.D	N.D	N.D
Motohiro et al. [29]	2	10	0	0	N.D	N.D	1.87 ± 1.43	0.12 ± 1.24
Merten et al. [30]	1	8	0	0	N.D	N.D	2.1 ± 2.6	−0.7 ± 2.8
Masuda et al. [31]	2	10	1	3	N.D	N.D	3.2 ± 3.3	0.5 ± 3.1
Maioli et al. [32]	25	29	1	1	N.D	N.D	N.D	N.D
PREVENT trial [33]	10	17	4	1	N.D	N.D	−0.49 ± 4.62	−2.58 ± 8.75
Koc et al. [34]	17	7	N.D	N.D	N.D	N.D	N.D	N.D
Klima et al. [35]	15	2	2	0	N.D	N.D	2.2 ± 2.7	−1.6 ± 2.3
Heguilén et al. [36]	3	6	N.D	N.D	N.D	N.D	N.D	N.D
Hafiz et al. [37]	14	19	N.D	N.D	N.D	N.D	N.D	N.D
Gomes et al. [38]	9	9	0	0	N.D	N.D	N.D	N.D
Castini et al. [39]	13	11	0	0	N.D	N.D	N.D	N.D
Briguori et al. [40]	2	11	1	1	N.D	N.D	N.D	N.D
Mahmoodi et al. [18]	12	34	N.D	N.D	−0.17 ± 0.02	0.08 ± 0.02	N.D	N.D

Author	CIN		Hemodialysis		Changes of serum levels of creatinine		Changes of serum levels of sodium bicarbonate	
	SB	SB + NAC	SB	SB + NAC	SB	SB + NAC	SB	SB + NAC

Sodium bicarbonate versus sodium bicarbonate plus N-acetyl cysteine								
Thayssen et al. (CINSTEMI trial) [22]	33	33	0	0	—	—	—	—
Yang et al. [20]	8	8	N.D	N.D	—	—	—	—
Ratcliffe et al. [17]	2	1	N.D	N.D	—	—	—	—
Heguilén et al. [36]	15	3	N.D	N.D	—	—	—	—
Heng et al. [41]	3	2	N.D	N.D	—	—	—	—

**(b):** 

Author	Changes of serum levels of potassium		Length of hospital stay		Mortality		MACE		Jadad
	SB	SC	SB	SC	SB	SC	SB	SC	
Sodium bicarbonate versus saline									
Boucek et al. [13]	N.D	N.D	8.4 ± 12.9	8 ± 10	0	0	3	3	4
Brar et al. [14]	N.D	N.D	N.D	N.D	3	3	4	8	4
Alessandri et al. [19]	N.D	N.D	N.D	N.D	N.D	N.D	N.D	N.D	3
REINFORCE trial [15]	N.D	N.D	N.D	N.D	N.D	N.D	N.D	N.D	5
Yang et al. [20]	N.D	N.D	N.D	N.D	N.D	N.D	N.D	N.D	3
Vasheghani-Farahani et al. [21]	N.D	N.D	1.5 ± 5	4.5 ± 3	N.D	N.D	N.D	N.D	5
Vasheghani-Farahani et al. [16]	N.D	N.D	1 ± 3	1 ± 6	N.D	N.D	N.D	N.D	5
CINSTEMI trial [22]	N.D	N.D	N.D	N.D	N.D	N.D	N.D	N.D	3
Ueda et al. [23]	N.D	N.D	22.8 ± 17.9	21.4 ± 19.6	2	8	9	10	4
Tamura et al. [24]	N.D	N.D	N.D	N.D	0	0	0	0	3
Shavit et al. [25]	N.D	N.D	N.D	N.D	N.D	N.D	N.D	N.D	2
RENO trial [26]	N.D	N.D	N.D	N.D	1	4	N.D	N.D	2
Ratcliffe et al. [17]	N.D	N.D	N.D	N.D	N.D	N.D	0	0	3
Pakfetrat et al. [27]	N.D	N.D	N.D	N.D	0	0	N.D	N.D	5
Ozcan et al. [28]	N.D	N.D	N.D	N.D	N.D	N.D	0	0	2
Motohiro et al. [29]	−0.46 ± 0.61	−0.32 ± 0.38	N.D	N.D	0	0	0	0	3
Merten et al. [30]	−0.26 ± 0.48	−0.17 ± 0.59	N.D	N.D	N.D	N.D	N.D	N.D	3
Masuda et al. [31]	N.D	N.D	N.D	N.D	0	2	N.D	N.D	3
Maioli et al. [32]	N.D	N.D	N.D	N.D	4	3	N.D	N.D	5
PREVENT trial [33]	−0.3 ± 0.58	−0.23 ± 0.52	N.D	N.D	0	1	10	3	5
Koc et al. [34]	N.D	N.D	N.D	N.D	N.D	N.D	N.D	N.D	2
Klima et al. [35]	N.D	N.D	N.D	N.D	5	4	N.D	N.D	4
Heguilén et al. [36]	N.D	N.D	N.D	N.D	N.D	N.D	N.D	N.D	3
Hafiz et al. [37]	N.D	N.D	N.D	N.D	0	0	0	0	3
Gomes et al. [38]	N.D	N.D	7.5 ± 10	8.6 ± 9.7	7	5	N.D	N.D	2
Castini et al. [39]	N.D	N.D	N.D	N.D	N.D	N.D	N.D	N.D	2
Briguori et al. [40]	N.D	N.D	N.D	N.D	N.D	N.D	N.D	N.D	3
Mahmoodi et al. [18]	N.D	N.D	N.D	N.D	0	0	N.D	N.D	2

Author	changes of serum levels of potassium		Length of hospital stay		Mortality		MACE		Jadad
	SB	SB + NAC	SB	SB + NAC	SB	SB + NAC	SB	SB + NAC	

Sodium bicarbonate versus sodium bicarbonate plus N-acetyl cysteine									
Thayssen et al. (CINSTEMI trial) [22]	—	—	—	—	—	—	6	3	3
Yang et al. [20]	—	—	—	—	—	—	N.D	N.D	3
Ratcliffe et al. [17]	—	—	—	—	—	—	0	0	3
Heguilén et al. [36]	—	—	—	—	—	—	N.D	N.D	3
Heng et al. [41]	—	—	—	—	—	—	N.D	N.D	3

**Table 3:** Subgroup analysis for clinical outcomes.

Subgroup	Studies (*N*)	Odd ratio or SMD (95% CI)	*P* value
S.G.A for CIN according to OR			
Age			
≤65	11	0.770 (0.582–1.019)	0.068
>65	16	0.765 (0.602–0.973)	0.029
Diabetic mellitus			
≤30	9	0.708 (0.535–0.937)	0.016
>30	14	0.774 (0.581–1.031)	0.080
Radiocontrast			
Iso-osmolality	9	0.902 (0.692–1.174)	0.442
Low-osmolality	18	0.663 (0.514–0.853)	0.001
Procedure			
Elective	23	0.869 (0.710–1.065)	0.176
Emergency	4	0.447 (0.290–0.687)	0.000

S.G.A for hemodialysis according to OR			
Age			
≤65	2	0.486 (0.088–2.697)	0.409
>65	8	1.177 (0.540–2.564)	0.681
Diabetic mellitus			
≤30	2	0.489 (0.088–2.702)	0.412
>30	7	0.918 (0.387–2.180)	0.847
Radiocontrast			
Iso-osmolality	4	2.459 (0.704–8.583)	0.158
Low-osmolality	6	0.594 (0.238–1.484)	0.265
Procedure			
Elective	8	1.425 (0.629–2.226)	0.396
Emergency	2	0.307 (0.060–1.572)	0.156

S.G.A for mortality according to OR			
Age			
≤65	2	0.885 (0.336–2.331)	0.804
>65	6	0.667 (0.341–1.303)	0.236
Diabetic mellitus			
≤30	4	0.681 (0.339–1.368)	0.280
>30	3	0.902 (0.352–2.311)	0.830
Radiocontrast			
Iso-osmolality	3	1.136 (0.444–2.906)	0.791
Low-osmolality	5	0.578 (0.290–1.151)	0.119
Procedure			
Elective	5	1.202 (0.618–2.339)	0.588
Emergency	3	0.199 (0.059–0.672)	0.009

S.G.A for length of hospital stay according to SMD			
Age			
≤65	4	−0.104 (−0.247 to 0.039)	0.153
>65	1	0.075 (−0.436 to 0.585)	0.774
Diabetic mellitus			
≤30	2	−0.240 (−0.526 to 0.047)	0.101
>30	3	−0.047 (−0.204 to 0.110)	0.560
Radiocontrast			
Iso-osmolality	All studies were low osmolality	All studies were low osmolality	All studies were low osmolality
Low-osmolality			
Procedure			
Elective	4	−0.104 (−0.247 to 0.039)	0.153
Emergency	1	0.075 (−0.436 to 0.585)	0.774

S.G.A for adverse events according to OR			
Age			
≤65	1	0966 (0.187–4.987)	0.961
>65	3	1.093 (0.574–2.081)	0.787
Diabetic mellitus			
≤30	1	0.817 (0.273–2.431)	0.713
>30	2	0.625 (0.238–1.643)	0.340
Radiocontrast			
Iso-osmolality	1	3.388 (0.918–12.509)	0.067
Low-osmolality	3	0.701 (0.340–1.443)	0.334
Procedure			
Elective	3	1.213 (0.590–2.490)	0.601
Emergency	1	0.841 (0.273–2.431)	0.713

S.G.A mean changes of serum creatinine to SMD			
Age			
≤65	2	−0.053 (−0.253 to 0.147)	0.605
>65	3	0.036 (−0.134 to 0.206)	0.677
Diabetic mellitus			
≤30	1	−0.156 (−0.397 to 0.085)	0.206
>30	4	0.062 (−0.092 to 0.215)	0.432
Radiocontrast			
Iso-osmolality	1	−0.156 (−0.397 to 0.085)	0.206
Low-osmolality	4	0.062 (−0.092 to 0.215)	0.432
Procedure			
Elective	All studies had elective procedure	All studies had elective procedure	All studies had elective procedure
Emergency			

S.G.A for mean changes of sodium bicarbonate according to SMD			
Age			
≤65	All studies had age more than 65 years	All studies had age more than 65 years	All studies had age more than 65 years
>65			
Diabetic mellitus			
≤30	—	—	—
>30	4	1.248 (1.058 to 1.439)	0.000
Radiocontrast			
Iso-osmolality	2	0.622 (0.449 to 0.795)	0.000
Low-osmolality	3	1.120 (0.889 to 1.352)	0.000
Procedure			
Elective	4	0.797 (0.654 to 0.941)	0.000
Emergency	1	0.843 (0.310 to 1.376)	0.002
