# Corrigendum to “Current Status of Sodium Bicarbonate in Coronary Angiography: An Updated Comprehensive Meta-Analysis and Systematic Review”

**DOI:** 10.1155/2018/7812708

**Published:** 2018-01-08

**Authors:** Sadeq Ali-Hasan-Al-Saegh, Seyed Jalil Mirhosseini, Elham Rahimizadeh, Zahra Ghodratipour, Zahra Sarrafan-Chaharsoughi, Ali Mohammad Dehghan, Mohammad Reza Lotfaliani, Mohammad Rezaeisadrabadi, Elham Kayvanpour, Farbod Sedaghat-Hamedani, Mohamed Zeriouh, Alexander Weymann, Anton Sabashnikov, Aron-Frederik Popov

**Affiliations:** ^1^Cardiovascular Research Center, Shahid Sadoughi University of Medical Sciences, Yazd, Iran; ^2^Department of Medicine III, University of Heidelberg, Heidelberg, Germany; ^3^Department of Cardiothoracic Transplantation and Mechanical Circulatory Support, Royal Brompton & Harefield NHS Foundation Trust, London, UK

In the article titled “Current Status of Sodium Bicarbonate in Coronary Angiography: An Updated Comprehensive Meta-Analysis and Systematic Review” [1], the name of the first author was given incorrectly as Sadegh Ali-Hassan-Sayegh. The author's name should have been written as Sadeq Ali-Hasan-Al-Saegh. The revised authors' list is shown above. The published spelling was the Persian transliteration, whereas the corrected spelling is the Arabic transliteration.
